# Housekeeping protein-coding genes interrogated with tissue and individual variations

**DOI:** 10.1038/s41598-024-63269-4

**Published:** 2024-05-30

**Authors:** Kuo-Feng Tung, Chao-Yu Pan, Wen-chang Lin

**Affiliations:** Institute of Biomedical Sciences, Academia Sinica, Taipei, 115 Taiwan, R.O.C.

**Keywords:** Protein-coding gene, Housekeeping genes, GTEx project, Next-generation sequencing, Gini index, Protein databases, Sequence annotation, Transcriptomics

## Abstract

Housekeeping protein-coding genes are stably expressed genes in cells and tissues that are thought to be engaged in fundamental cellular biological functions. They are often utilized as normalization references in molecular biology research and are especially important in integrated bioinformatic investigations. Prior studies have examined human housekeeping protein-coding genes by analyzing various gene expression datasets. The inclusion of different tissue types significantly impacted the discovery of housekeeping genes. In this report, we investigated particularly individual human subject expression differences in protein-coding genes across different tissue types. We used GTEx V8 gene expression datasets obtained from more than 16,000 human normal tissue samples. Furthermore, the Gini index is utilized to investigate the expression variations of protein-coding genes between tissue and individual donor subjects. Housekeeping protein-coding genes found using Gini index profiles may vary depending on the tissue subtypes investigated, particularly given the diverse sample size collections across the GTEx tissue subtypes. We subsequently selected major tissues and identified subsets of housekeeping genes with stable expression levels among human donors within those tissues. In this work, we provide alternative sets of housekeeping protein-coding genes that show more consistent expression patterns in human subjects across major solid organs. Weblink: https://hpsv.ibms.sinica.edu.tw.

## Introduction

The rapid progress of next-generation sequencing technologies has led to the accumulation of large-scale sequencing datasets through diverse genomic and transcriptome research endeavors^1^. These studies offer novel perspectives on the genomic characteristics and regulatory mechanisms of functional protein-coding genes in various organisms^2–4^. Furthermore, the generation of additional gene expression datasets through the expansion of single-cell RNA-seq projects contributes to the advancement of new understanding of cell-type specific modulation expressions on protein-coding genes^5,6^. This knowledge is crucial for the understanding of biological functions for protein-coding genes. Previous studies have employed the GTEx gene expression data to investigate the tissue-specific expression patterns of human protein-coding gene transcripts^7,8^. It is noteworthy that a greater number of protein-coding gene transcripts exhibit selective tissue modulations than initially expected. In addition, the majority of tissue expression profiles exhibit a correlation with their distinct biological roles^9,10^. Nevertheless, it has been observed that a considerable number of protein-coding genes undergo distinct modifications in different tissues; whereas selected protein-coding genes nevertheless exhibit consistent expression patterns across many tissue types.

Interestingly, these genes that are uniformly expressed have been previously categorized as housekeeping genes in the past^11^. While several prior research studies have examined these so-called housekeeping genes^11–14^, a decisive and comprehensive description on the housekeeping gene has yet to be settled. Commonly, housekeeping genes are usually acknowledged as genes that exhibit general expression across many tissue and cell types in organisms. One of the essential characteristics of housekeeping genes is their noticeable expression across all tissue types analyzed^11,15^. This statement should be amended to provide a comprehensive analysis of numerous tissue types, incorporating a multitude of transcriptome research. Furthermore, it is suggested that apart from the commonly seen expression, additional characteristics should be employed for housekeeping genes^14^, including the implementation of a minimum expression level cutoff. In certain research endeavors, housekeeping genes could also refer to as the indispensable collection of essential genes responsible for upholding cellular survival functions^12^. However, the inclusion of the essentiality feature as housekeeping genes is a subject of debate. It is our contention that essential genes ought to be delineated according to more precise biological criteria.

Previous studies utilizing cDNA libraries and EST platforms encountered limitations in the scope of housekeeping genes investigated^16^. The utilization of expanded next-generation sequencing datasets enables the observation of a greater number of genes expressed across diverse tissue types^12^. Additionally, the quantification of gene expression levels is influenced by the depth of sequencing. With the progression of next-generation sequencing datasets, there is an anticipated improvement in the comprehensiveness and understanding of gene expression across a wider range of tissue types. Given the increasing availability of transcriptome datasets and the expanded diversity of tissue types, it is becoming apparent that broad expression profiles across all tissue types alone may not be appropriate to identify housekeeping genes^11^. Indeed, this observation is particularly true for the numbers of diverse tissues investigated for a given study. This would be more sever with increasing single cell transcriptome analyses to cover even greater cell types^17,18^. It is anticipated that the amounts of housekeeping genes can vary from a few hundred to several thousand genes, depending on the numbers of tissue samples; specific criteria and experimental settings^14^. Besides the expression status, consistent expression levels and statistical variations were also considered for housekeeping genes among the datasets in several studies^11,13,19^. Thus, different statistical techniques were employed to identify housekeeping genes, such as expression levels, tissue-specific index, weighted expression intensity, coefficient of variation, Z-score, Preferential Expression Measure, Gini index, Kendall Tau, among others^20–22^. Thus, it is no surprise that there exist diverse numbers of investigation findings that compile lists of housekeeping genes; yet, a universally consensus collection of housekeeping genes has yet to be generated.

Moreover, the majority of relevant investigations have focused on examining the association in average gene expression between major tissue types exclusively. There is a lack of comprehensive research considering both the human subject variations and the tissue profiles for housekeeping genes. Insufficient study has been conducted on tissue expression profiles and individual sample variances among human subjects. Hence, doing a more comprehensive analysis of the expression profiles of housekeeping genes would be advantageous. We are interested to expand interrogation the housekeeping protein-coding genes with GTEx datasets. GTEx project is a well-known project, which provides non-cancerous normal tissue expression profiles on human genes in numbers of individual human subjects^7,23^. GTEx project provides not only significant numbers of various tissues as well as individual clinical subjects, which allows us to perform more investigation on the stably expressed housekeeping protein-coding genes.

## Results

### Protein-coding genes in normal tissue subtypes

**Figure 1:**
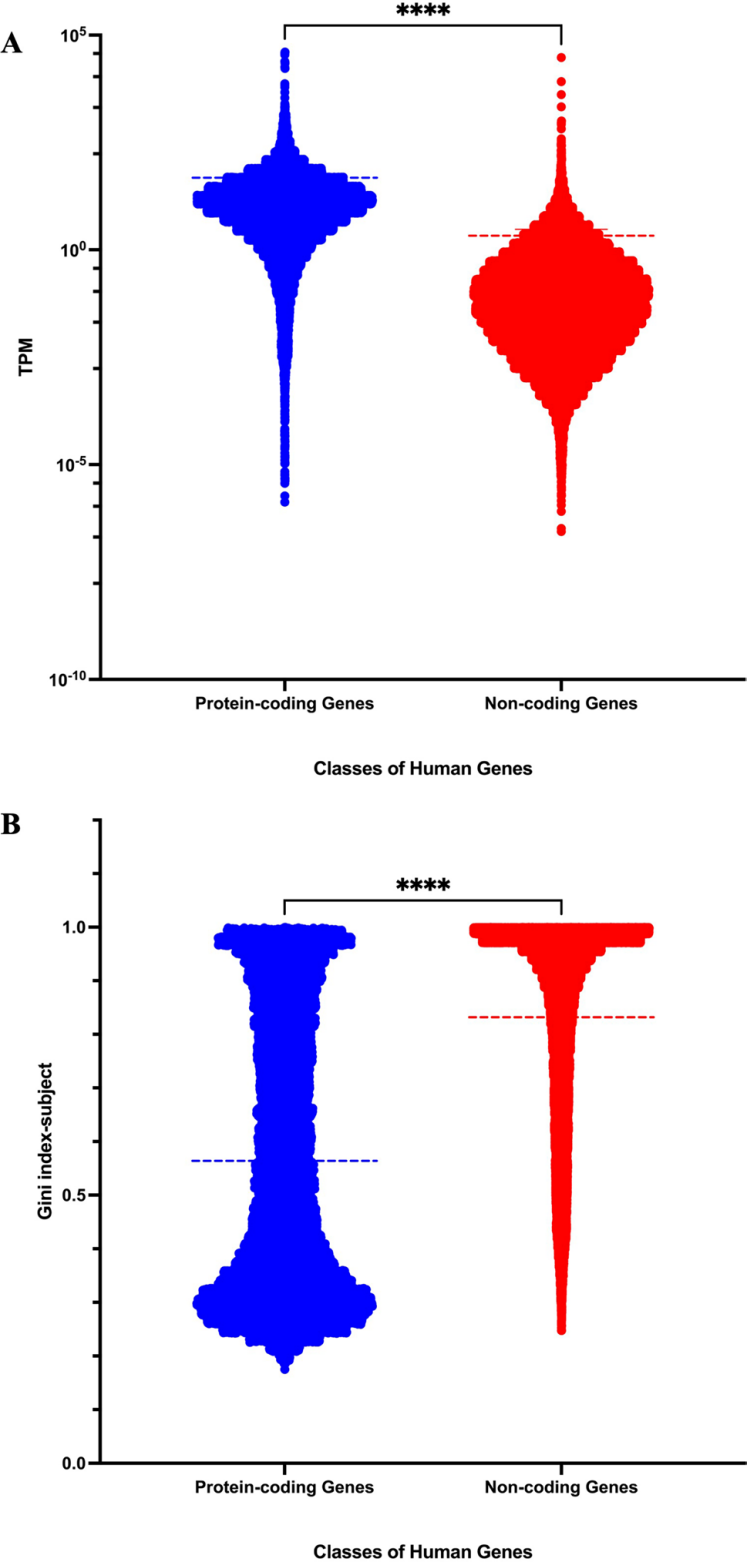
(**A**) Average TPM expression distribution of human protein-coding genes and non-coding genes. The mean TPM value of 19,273 protein-coding genes is 47.78; mean TPM value of 36,883 non-coding genes is 2.14. (**B**) Gini index-subject distribution of human protein-coding genes and non-coding genes. The mean Gini index-subject value of protein-coding genes is 0.563; mean Gini index-subject value of non-coding genes is 0.831. Unpaired t-test was conducted ****P-value < 0.0001.

In this report, we would like to focus more on the interrogation of those stably expressed protein-coding genes in normal human tissues with GTEx datasets. Recently, our laboratory also examined the expression of transcript isoforms of human protein-coding genes using the GTEx datasets^9,10,24^. Herein, we retrieved mainly the gene expression level datasets of 54 GTEx tissue subtypes. Two cell lines datasets were excluded in the beginning and we used only the normal tissue expression information. In brief summary, there are 56,200 gene expression information in total with 52 tissue subtypes obtained from 16,704 GTEx individual samples. Initially, we used all 16,704 samples obtained from 52 different tissues to calculate the Gini index, which measures the diversity in gene expression among all human subjects (referred to as Gini index-subject). Subsequently, identical gene ID records with duplicated chromosome locations were noted and we removed those 44 records containing “PAR-Y” chromosome locations. Among the remaining 56,156 human genes, we specifically focused on the 19,273 protein-coding genes based on their GENCODE biotype feature (protein-coding). As reported previously^10,24^, protein-coding genes have higher expression levels (mean TPM 47.78; Fig. 1A), and more ubiquitous expression profiles among 16,704 samples (average Gini index-subject value 0.563; Fig. 1B). Thus, there are remaining 36,883 non-coding genes in the GTEx dataset. Those non-coding genes do have lower expression levels and more diverse expression profiles (mean TPM 2.14; average Gini index-subject 0.831). This should be attributed mostly to the excessive sample variation resulted from very small expression values in most of non-coding genes, including no expression values for many genes. It is likely that not all genes were expressed in the GTEx datasets. Even within the protein-coding genes, there are 32 genes without expression values among the 52 tissue subtypes analyzed here.

### Gini index measurement for normal tissue expression profiles

In this report, in order to interrogate putative stably expressed housekeeping protein-coding genes, we utilized the Gini index approach which was first applied for housekeeping gene studies by O’Hagan et al.^22^. Gini index was first utilized for measuring economical income inequality by Dr. Corrado Gini, and it has been applied to study the distribution inequality nature in various research fields^25^. The Gini index is a non-parametric metric used in economics to describe the income inequality within a community. In 2016, Jiang et al. adapted the Gini index to identify rare cell type-associated genes^26^. Subsequently, O’Hagan et al. utilized the Gini index for classifying housekeeping genes using average TPM values of various tissue types^22^. It measures inequality on a scale from 0 to 1, where higher values indicate higher variations. Therefore, a Gini index value of zero would indicate perfect equality. We believe that this is a suitable measurement tool for stable gene expression profiles. In previous reports, housekeeping genes were defined with the Gini index values less then 0.2, which would be utilized in this study^22^.

**Figure 2:**
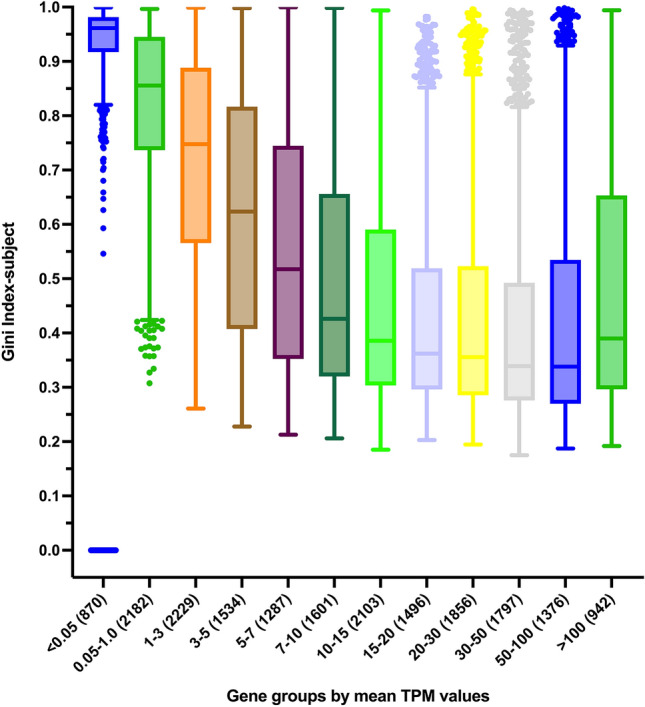
Distribution of Gini index-subject among diverse expression groups of protein-coding genes. Human protein-coding genes are categorized into 12 groups based on their expression levels. A box-and-whisker plot is used to visually represent the distribution of the Gini index-subject among respective groups of protein-coding genes. The TPM expression range for each group is labeled, and the number of genes in each group is shown in parentheses.

We started with the 19,273 protein-coding genes. Since housekeeping genes are generally defined to have ubiquitous expression profiles among tissues at first place, it is also noted that the numbers of sample without expression value would have influence on the Gini index determination, thus, the none and lowly expressed protein-coding genes would be excluded for the housekeeping gene examination here. We then used 0.05 TPM as the housekeeping gene cutoff criterion in this report, and there are remaining 18,403 genes above 0.05 TPM (Fig. 2, following lowly expressed 870 genes removed). This summarized data also demonstrated that abundantly expressed protein-coding genes do have more general expression profile among different tissue types. It is interesting to note that the highly expressed group (TPM values more than 100) does have slightly higher Gini index values (Fig. 2), which could imply certain protein-coding genes with considerable tissue specific expression profiles.

### Gini index by individual subjects and tissue subtypes

Previously in most reports, the Gini index is mostly calculated with average tissue expression values in the datasets for examining tissue variations^22,25^. Herein, we are interested in the human subject variations in addition to the tissue variations for stably expressed housekeeping protein-coding genes. Besides the 52 tissue subtypes difference, we would like to further interrogate the expression profiles among all individual subjects. As described earlier, we first used all 16,704 samples from 52 tissue subtypes to obtain Gini index-subject values for each protein-coding gene. However, we did notice that there is significant sample size difference among the 52 tissue subtypes (Supplementary Table 1). Muscle_skeletal has the greatest number of samples (803); on the contrary, kidney_medulla contains only 4 samples. For some major organs, there are additional subtypes or physiological tissue locations collected. For instance, brain has 13 subtypes with one to two hundred samples in each subtype and it would add up to 2642 samples collected in total from the brain organ (15.8% of all GTEx samples). Esophagus, artery, skin and adipose all have combined case numbers over 1000 samples. On the other hands, some organs have less than 100 samples collected (bladder, cervix, fallopian_tube, kidney).

**Figure 3:**
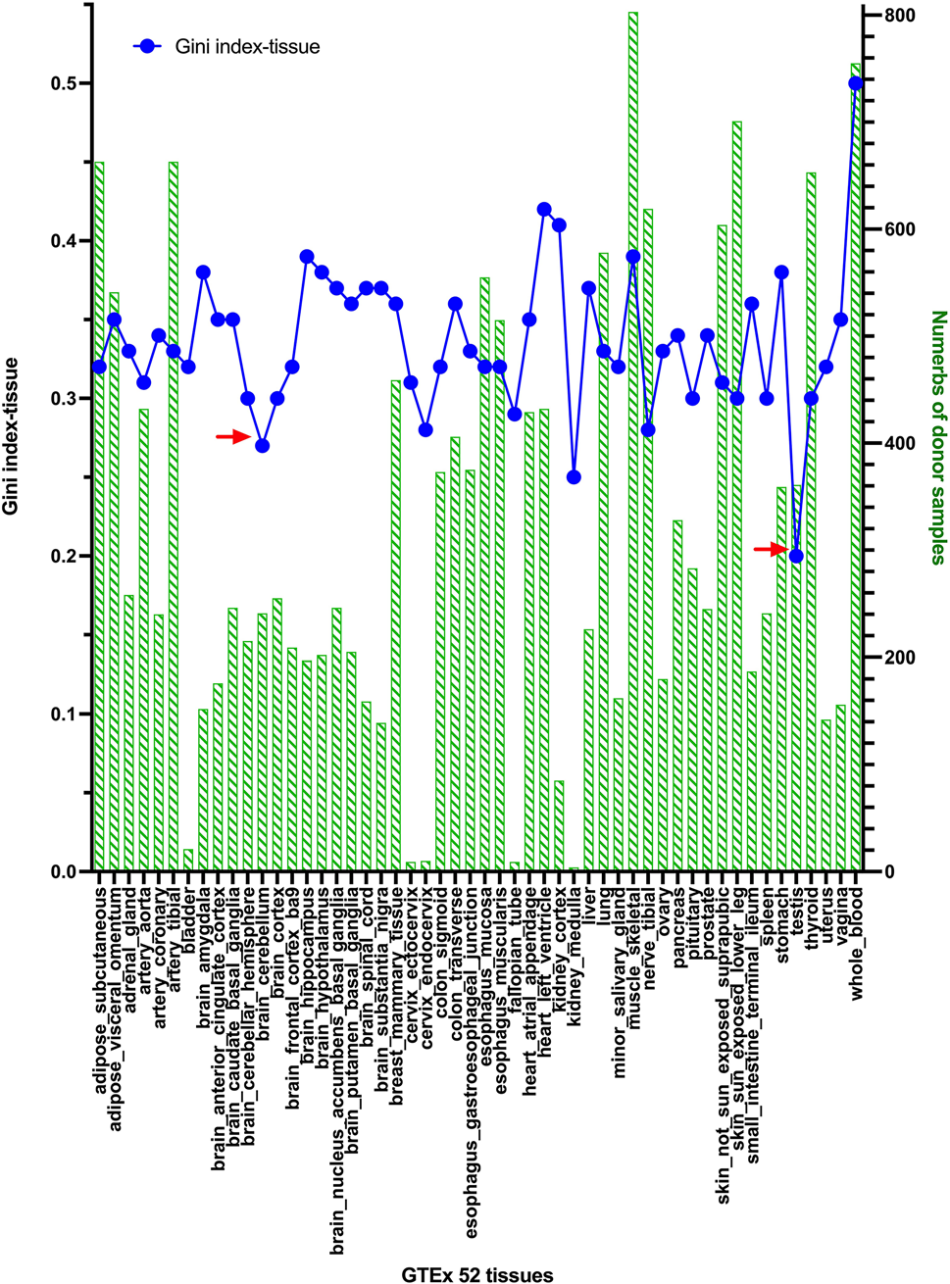
Illustration of Gini index-tissue values and numbers of donor samples in 52 tissue subtypes. We calculated the Gini index-tissue values for each specific tissue dataset by utilizing the donor samples within each tissue subtype. The average Gini index-tissue values for all protein-coding genes were calculated and displayed as the blue symbols. The donor samples for each tissue subtypes are shown by light green bar graphs. Please note that the cerebellum and testis tissues have relatively low Gini index-tissue values, as indicated by the red arrows.

Therefore, we further inspect the gene expression profiles within separated particular tissue subtypes (referred to as Gini index-tissue). We calculated the Gini index-tissue values of protein-coding genes among individual samples within each tissue subtypes, instead of all 16,704 samples combined from 52 tissue subtypes (Supplementary Table 1). In Fig. 3, the average Gini index-tissue values of all protein-coding genes and the tissue sample size for particular tissues are illustrated. Besides some tissue subtypes with small sample sizes having expected lower Gini index-tissue values, two interesting tissue subtypes with relative low average Gini index-tissue values are cerebellum and testis (Fig. 3, red arrows). On the contrary, blood has the highest average Gini index-tissue value (0.5) than all other tissues, which might implicate more heterogeneities in diverse haemopoietic cell compositions and immune status among human subjects, unlike other solid tissues. However, this observation should be further interrogated in more details in the future.

**Figure 4:**
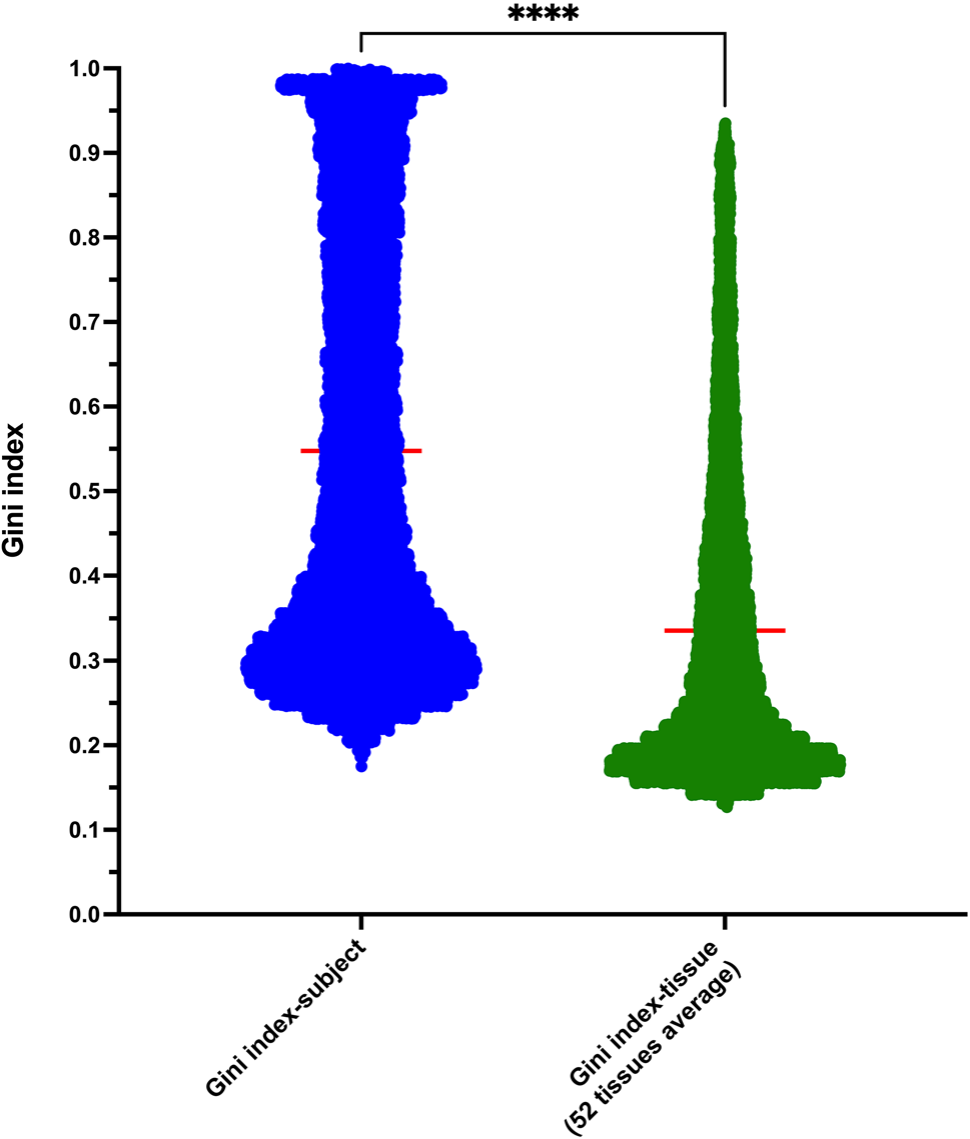
Different types of Gini index distribution of human protein-coding genes. The scatter plot demonstrates Gini index-subject values (blue color) and average Gini index-tissue values (green color) for protein-coding genes. The Gini index-subject value is calculated by combining 16,704 samples of all tissues. The Gini index-tissue values were determined using samples from each tissue subtype, and the average Gini index-tissue value from 52 tissues was then obtained. Paired t-test was conducted. ****P-value < 0.0001.

It is reasonable that the donor samples within the same tissue subtype would have similar expression profiles and one expect to observe lower Gini index-tissue values. Combined samples from more different tissue types should have increased cell type composition and be anticipated to have larger Gini index values. In Fig. 4 for all protein-coding genes, the average of separate Gini index-tissue values from 52 tissues are much lower comparing to the Gini index-subject calculated from combined 16,704 samples (0.335 vs 0.547). In this respect, the tissue subtypes restricted expression profile will not be represented by using the Gini index-tissue values obtained from studied tissues. Therefore, we then tried to calculate the Gini index using the average expression TPM values of 52 tissue subtypes as performed in most previous studies (referred to as Gini index-TPM). It is no surprise that the Gini index values increased due to the tissue variations, however, it is still less than the values obtained from 16,704 samples (Gini index-subject; Supplementary Fig. 1), which would consider also the individual sample variations.

In order to further identify the housekeeping genes are stably expressed in all human subject samples, we initially selected the genes with their Gini index-subject values less than 0.2 as defined in previous reports. There are twenty housekeeping genes using the GTEx dataset with combined 16,704 sample subjects from 52 tissues (Table 1). The average Gini index-subject value of these 20 genes is 0.192. We further used the DAVID pathway analysis tool for their functional annotation. For these 20 genes, they are enriched for mitochondrion, TORC1 signaling as well as energy homoeostasis modulations. When we further inspected their expression profiles in separate tissue subtypes, these 20 genes also have the low Gini index-tissue values in most tissue subtype, but not all 52 tissue subtypes (Table 1). This suggests that there are indeed tissue subtype variations as discussed, especially affected by the sample sizes of certain tissue subtypes.

**Table 1:** Housekeeping genes defined with Gini index-subject values.

Symbol of protein-coding genes	Gini index-subject from 16,704 subjects	Average TPM values of gene expression	Counts of tissue with Gini index-tissue ≤ 0.2
SSU72	0.188	43.933	42
LAMTOR5	0.192	82.343	40
POLR2J	0.175	44.403	47
AP3S2	0.185	14.593	39
SDHC	0.186	13.031	43
NDUFA2	0.186	48.747	42
BABAM1	0.187	63.063	40
TXN2	0.188	67.817	47
DNAJA2	0.190	34.891	38
TUFM	0.192	135.955	43
MAP2K2	0.193	87.533	42
LAMTOR2	0.194	33.766	41
CINP	0.194	12.177	41
TIMM17B	0.195	29.321	40
NEDD8	0.196	49.211	40
RBX1	0.196	69.792	42
C1orf43	0.197	112.599	39
CLPP	0.197	57.433	40
IDH3G	0.197	52.617	38
EDF1	0.200	260.812	43

The Gini index-subject were determined with 16,704 subject samples from all 52 tissue subtypes. We selected protein-coding genes with the Gini index-subject values less than 0.2. On the other hands, the Gini index-tissue was computed based on individual samples within each subtype of tissue for 52 tissue subtypes.

### Housekeeping genes defined by Gini index-tissue value within tissues

As mentioned, there are different tissue subtypes and varied sample numbers in each subtype. If the housekeeping gene cut-off criteria is set to less than 0.2 Gini index value, we could observe extreme distribution among each 52 tissue subtypes (Supplementary Table 1). Testis and cerebellum have more that 10,000 genes with their Gini index-tissue value less than 0.2. Some tissue subtypes have less than 100 defined housekeeping genes, including the blood tissue mentioned earlier (only 35 genes). This raises one interesting issue for housekeeping gene determination with combined samples from various tissues. Nonetheless, it provides an initial assessment for the universal gene expression patterns among the subject samples interrogated. Additional considerations might take into account. Besides the individual donor variations (sex, age, health status etc.), there are many possible conditions would contribute to the variations, such as sample size, very low gene expression level, tissue subtype locations heterogeneities during sample collections. Intriguingly, particular observations were found within different location subtypes of same major organs, such as brain_cerebellum (10,525) and brain_hippocampus (56); heart_atrial_appendage (4662) and heart_left_ventricle (19); kidney_cortex (13) and kidney_medulla (8702). While there is still sample size consideration (kidney, for example), it is possible that one arbitrary cutoff criterion of Gini index value is not suitable for diverse tissue subtypes. Different tissue subtypes might need to have different housekeeping gene collections for subsequent suitable interrogations, especially integrated systems biology studies would include human disease samples (such as TCGA datasets for cancer biomarker studies).

We attempted to look for low Gini index-tissue based housekeeping genes in maximum overlapping tissue subtypes. We used Gini index-tissue value less than 0.2 as the cut off for housekeeping genes in each tissue subtype. As shown in Supplementary Table 1, there are large difference among tissue subtypes with the numbers of housekeeping genes having Gini index-tissue value less than 0.2 in each tissue. For each protein-coding gene, we further examined the count numbers of tissues with their Gini index-tissue value less than 0.2. There are only 4 genes identified here with at most 49 tissue subtypes. They are SHARPIN; TMEM219; ZNF768 and CTDNEP1. Intriguingly, the Gini index-subject values of these four genes determined from 16,704 combined samples are from 0.217 to 0.273. This suggest that more homogeneous expression profiles within separate tissue subtype for these protein-coding genes. This would also imply the sample number variation in tissue subtypes from GTEx datasets. It is noted that there are 7 genes with following maximum 48 tissue subtypes, USF2, RPS15, ECI1, CARS2, LAMTOR1, RBM17, RCC1L.

Furthermore, one would expect that there are greatly similar gene expression profiles with different tissue subtypes from the same organ type. In the adipose tissues, there are 5,735 overlapping housekeeping genes between adipose_subcutaneous and adipose_visceral_omentum. There are 6263 overlapping housekeeping genes among the three esophagus subtypes. With increased tissue subtypes, we would expect more diverse tissue composition and biological variations. For further investigations, we used the diverse 13 brain subtypes and tried to identified overlapping housekeeping genes among them. The numbers of identified genes reduced to 26 genes; and their average Gini index-tissue value is 0.29. However, another question is the general gene expression levels in certain brain subtypes. There are several brain subtypes with low numbers of housekeeping genes due to possibly very low gene expression levels for most protein-coding genes. The numbers of samples in different tissue subtypes would also have impacts on the calculation of Gini index. Thus, selection of tissue subtypes would have strong influence on the housekeeping genes identified in certain studies.

### Selected representative tissue subtypes for housekeeping genes

Finally, in order to establish a putative more representative tissue housekeeping genes among major organs, we then removed some tissues with small numbers of samples and also tried to select one representative tissue subtype for each major organ. We then chose 27 tissue subtypes and calculated the Gini index-subject again with 9943 individual samples from this 27 tissue-subset (Methods). Herein, the protein-coding genes analyzed are 18,439 genes with again the TPM cutoff at 0.05, since the gene expression average is changed. This 27 tissue-subset would provide better interrogation on the consensus housekeeping genes for human subject variations and major human organs. Lastly, we discovered 335 housekeeping protein-coding genes (Gini index-subject value less than 0.2 from 9943 subjects) using this 27 tissue sample subset (Supplementary Data file). Again, not all 335 genes have their tissue subtype Gini index-tissue less than 0.2. For example, one of the gene, CHCHD4 with the Gini index-subject value of 0.192, but only 21 out 27 tissues have the Gini index-tissue less than 0.2 for this gene. This implied that CHCHD4 expression profile is indeed varied in some tissues (bladder, liver, lung, stomach etc.). We then tried to use the housekeeping genes identify separately in each tissue subtypes and search for housekeeping genes conserved in all 27 tissue subtypes. There are 529 genes identified for such tissue-oriented housekeeping genes using Gini index-tissue cutoff (Supplementary Data file). In this group, these housekeeping genes have their Gini index-tissue less than 0.2 within all 27 tissue subtypes respectively, but they do still have substantial tissue variations between tissues for some genes. There are 411 genes have their sample Gini index-subject value above 0.2 (calculated with all 9943 donor tissue samples). The highest one is MRGBP gene, which have a very high expression profile in testis tissue only (it is noted that such a tissue expression profile by individual genes could be observed using our new bioinformatic web tool, https://hpsv.ibms.sinica.edu.tw). In addition, relatively higher tau and CV values are observed with these genes (not shown). Due to the sample number concerns, we think the combined strategy with overlapping housekeeping genes might be beneficial for understanding the tissue expression profiles of protein-coding genes. In summary, these represented 118 genes intersected between the 335 (Gini index-subject) and 529 (Gini index-tissue) housekeeping gene lists might be a useful stably expressed housekeeping gene collection for future housekeeping protein-coding gene research applications (Supplementary Data file). These housekeeping genes are enriched in the mitochondria and ribosome functions as implicated by the DAVID analysis webtool.

Extensive research has been conducted using the GTEx datasets, including gender differences in gene regulation^27,28^. Some protein-coding genes and transcription factor genes have gender-specific effects on the regulation of gene expression and the presence of expression quantitative trait loci, namely PZP and VWCE genes expression in liver tissue^28^. We also investigate how gender influences protein-coding gene expression differences between male and female groups. It is important to clarify that we are not comparing the differential expression as in earlier research; the Gini index values used here indicate the variation between individual male or female subjects in each group. Therefore, it is anticipated that the majority of protein-coding genes exhibit minimal disparity between the Gini index-tissue for males and the Gini index-tissue for females. Nevertheless, we did observe variations in the PZP and VWCE genes inside the liver tissue. The Gini index-tissue (male) is 0.65 and the Gini index-tissue (female) is 0.41 for PZP gene. Conversely, for VWCE gene, the Gini index-tissue (male) is 0.43 and the Gini index-tissue (female) is 0.55. To facilitate the exploration of Gini index-subject and Gini index-tissue profiles and gene expression patterns in various human tissues, we have developed a user-friendly bioinformatic web tool. This tool allows for the visual presentation of important information for each protein-coding gene; including additional Gini index-tissue (female) and Gini index-tissue (male), as illustrated in Supplementary Fig. 2. This web database can be accessed through: https://hpsv.ibms.sinica.edu.tw.

## Discussion

Housekeeping genes are often used to standardize and normalize gene expression results in a variety of biological experimental settings. They are thought to be essential for cellular viability and remain active independent of their biological roles in diverse tissues^12^. In general, housekeeping genes remain stable expression levels regardless of tissue type, and developmental stage. Later studies emphasize steady and consistent expression rather than using a uniform expression threshold across tissue types studied^14^. Furthermore, experimental verification in clinical samples would be necessary. Significant researches have used large numbers of mass spectrometry datasets and TCGA cancer transcriptome datasets for novel housekeeping reference genes and proved the efficacy of alternative reference genes after molecular biology experimental validations^29,30^. Dr. Park’s prominent results indicate that the commonly utilized control genes were not deemed the most reliable housekeeping genes based on PCR and western blotting investigations^29,30^, and this was also reiterated by others^17,25^. In our findings, these well-known housekeeping control genes have greater Gini index-subject values, with over 10,000 GTEx human subjects assessed. (GAPDH: Gini index-subject 0.427; ACTB: Gini index-subject 0.428; HPRT1: Gini index-subject 0.342; B2M: Gini index-subject 0.418). Although housekeeping protein-coding genes are frequently not the primary subjects of investigation, it is still necessary to possess comprehensive lists of such genes in normal tissues in order to perform differential gene expression analyses that aim to identify human disease biomarkers, which is more important in systems biology studies. We conducted a comparative analysis of our housekeeping gene findings with those from the well-known human normal tissue database, Human Protein Atlas^31^. The 335 housekeeping reference genes (Gini index-subject) had an average HPA Gini index value of 0.20 and an average CV value of “37.6%” in the HPA dataset. The average CV value for all genes in the HPA dataset is “172.7%”. The GTEx findings and HPA dataset show a strong concurrence. In contrast, the TCGA databases include a very small number of normal samples (hundreds), while including over 20,000 cancer tissues^29,32^. There is a requirement for valuable housekeeping reference genes derived from normal tissues to be useful in TCGA cancer biomarker studies. We hope that our work, which identified stably expressed housekeeping genes in normal tissue subtypes using a large number of GTEx human participants, may be useful for future personalized cancer biomarker investigations.

The GTEx project was created for human genetic variants and, in particular, for unique eQTL analysis, which provides one of the most useful datasets for studying human genome variations on gene expressions^7,23^. Recently, our laboratory has used these critical transcriptome data resources to provide tissue expression profiles for human reference MANE-select transcripts^9^. Furthermore, it has been a vital and remarkable source for investigations into human gene expression in normal tissues^6,8,33–35^, which also included long-read and single-cell NGS studies for normal tissues in large numbers of human subjects. It is difficult to create such gene expression databases in human normal tissue samples, especially with a large number of contributors. Specifically, utilizing the GTEx transcriptome datasets, numerous fascinating findings investigated gene expression modulations across normal tissues in human subjects impacted by age and sex^27,28,36,37^. These reports implicated gene expression variations for human individuals. Some of the age-associated genes identified had higher Gini index values, reflecting gene expression modulations in human subjects across age groups, such as RSPO1 (Gini index-subject: 0.791); LTBP2 (Gini index-subject: 0.715); ALOX15B (Gini index-subject: 0.795). This finding lends validity to the Gini index’s use in evaluating gene expression differences in human samples. While the Gini index is a non-parametric measure commonly used to assess economic inequality within a community or country, it appears to be useful for analyzing gene expression information from various experimental sources. In a benchmark review publication for studying tissue specific genes^21^, it suggests that the both tissue specificity index (Tau) and Gini index show minimal variation beyond the genes that are most distinctive to a particular tissue. In our preliminary tests, we discovered that the Gini index correlates better with C.V. values for the finding of general expression housekeeping genes. Furthermore, large sample sizes appear to affect Tau profile determinations (the median number of Tau calculated is 0.930 and it is 0.489 for Gini index-subject with over 10,000 GTEx subjects). The capability of managing undetectable expression values for many protein-coding genes is yet another advantage of the Gini index computation^22^. In this research, we focus on 16,704 GTEx samples from normal human tissues and investigate their importance across human subjects. Although there is less interest in persistently expressed protein-coding genes, in this paper, we are interested in individual differences in the expression profiles of stably expressed housekeeping protein-coding genes. While most previous research looked at summarized tissue expression data from fewer than dozens of tissue types^11,16,20,22^, we examined the stably expressed housekeeping genes using a large number of all GTEx human subjects (by Gini index-subject) as well as the human subject variations within each tissue subtype (by Gini index-tissue). Our findings provide a complete investigation of the stable expression profiles of housekeeping genes in all GTEx participants, as well as an examination of human variations within each tissue subtype.

Molecular biological investigations, such as qPCR or western blotting, are crucial laboratory techniques for confirming the expression of protein-coding genes in clinical samples. Identifying these consistently expressed housekeeping genes is essential for accurately measuring gene expression levels and sample normalization comparison. Given the constraints of the instrument and technological capabilities, it is preferable to employ reference housekeeping genes that have a greater expression level. The mean TPM levels for frequently used protein-coding genes such as GAPDH are often higher than 45 TPM. In a prior comprehensive investigation^29^, a total of 38 novel discovered reference genes were found and confirmed to be suitable for cancer research purposes. The expression levels of these reference genes range from 8 to 737 TPM, with an average value of 115^29^. Therefore, it is advisable to utilize housekeeping genes that are highly expressed for experimental references. Among the 335 and 529 potential housekeeping genes identified in this study, a majority (75–80%) have high expression levels with TPM values over 20. It is recommended to pick genes with a TPM value over 20 when working with GTEx datasets. However, there are additional factors to consider when quantifying protein expression by western blotting assays, as the effectiveness of protein binding might vary greatly between assorted antibodies^30^. Besides, the quantity of mRNA transcripts is not necessarily correlated with the number of proteins following post-translational modifications. Additional proteome investigations are strongly advised for undertaking protein expression assays^30^, whereas the reference housekeeping genes reported here should be seen as promising preliminary western blotting options.

In general, the housekeeping genes were first regarded by their expression status (all or none) across different tissue types studied, which were constrained by the technological platforms (ESTs and even microarray) in previous findings^16,38^. With the growth of NGS transcriptome datasets, the number of human housekeeping genes found expanded significantly (from hundreds to thousands)^11,38^. With 16 normal tissues (transcriptome datasets of Human Body Map), 3804 possible housekeeping genes were found^11^. The majority of the housekeeping genes mentioned in the paper are included in the Eisenberg research^11^, namely 307 out of 335 genes and 426 out of 529 genes. Compared to the 115 GeneGini genes described in a significant paper utilizing the Gini index^25^, which identified those housekeeping reference genes overlapped across CCLE, HPA, and Klijn transcriptome datasets (mainly cancer cell lines). There are 56 GeneGini genes in common with our findings with GTEx normal tissues. In this paper, we examined the variability in human subjects within normal tissue subtype (based on Gini index-tissue) as well as the expression of consistently stable housekeeping genes in a significant number of all GTEx human subjects (based on Gini index-subject). Users can gain further knowledge on the various characteristics of housekeeping genes among tissue subtypes. It is widely acknowledged that the selection of housekeeping genes may vary depending on the dataset and the specific types of tissue being studied, as discussed in papers^14,25^. We concluded that the most important element may be the number of tissue types used in all investigations, as the majority of the studies investigated the average expression levels of genes in obtained tissue types. It is not surprising that kinds and numbers of tissues would influence the housekeeping genes revealed in respective investigations, resulting in a smaller consensus list of housekeeping genes with more different tissue types included, in addition to gene annotation consistency issues in different publications. As seen by the growing number of single-cell NGS transcriptome investigations, there are more different cell types and complex cellular gene expression patterns revealed than expected.

A recent significant work on circadian related human protein-coding gene expression utilizing GTEx datasets found tissue dissimilarity in gene expression rhythms on chosen gene groups^36^. This suggested that unique tissue-specific housekeeping genes would be more suited for certain research types. According to our findings, certain genes have a low Gini index-subject but not necessarily a lower Gini index-tissue in all tissue types. In a previous report^25^, three genes (CHMP2A, VPS29, and PCBP1) had an extremely low Gini index in both the tissue and cell line datasets evaluated. Intriguingly, with the study on 27 main tissue types here, CHMP2A has a Gini index-subject value of 0.173 in all samples, however only 26 of the 27 tissues have a Gini index-tissue value of less than 0.2. The Gini index for the VPS29 gene is 0.195, and 25 of 27 tissues have the Gini index-tissue values less than 0.2. Furthermore, PCBP1 has only 20 tissues with Gini index-tissue values less than 0.2. This implied the human individual subject variations for these genes in some tissue subtypes. Another example is the PRDX1 gene, which encodes a peroxiredoxin antioxidant enzyme. It was shown to be the sole common housekeeping gene in 15 datasets evaluated in one study^14^. PRDX1 has a Gini index-subject value of 0.306 and an average Gini index-tissue value of 0.181, as estimated using the dataset of 27 major tissues. However, only 19 of the 27 tissues had a Gini index-tissue of less than 0.2 for the PRDX1 gene. This implied that PRDX1 is consistently expressed throughout 27 tissue types, however expression levels vary between individual donors in each tissue type.

Among the Gini index-tissue distributions evaluated with 27 major tissue types, liver, muscle_skeletal, and stomach had the highest variances and the fewest number of housekeeping genes. It is possible that these organs might show more variations and reactions to environmental or nutritional factors. On the other hand, as stated in the result section, testis and cerebellum tissues have a higher number of housekeeping genes (defined by Gini index-tissue less than 0.2). Interestingly, there are research and review publications that describe comparable gene expression patterns in the testis and cerebellum^39–41^. Although these two organs are totally different tissue types, they have some shared molecular properties and similar proteins involved in exocytosis, signaling processes, and tissue growth. It is intriguing that there is less individual variation in the gene expression patterns among GTEx samples in these two tissue subtypes. In summary, we provide alternative sets of housekeeping protein-coding genes in this study. These sets exhibit expression patterns in human subjects that are more consistent across major solid organs.

## Methods

### GTEx V8 and HPA datasets

The GTEx (Genotype-Tissue Expression) Project is an excellent resource for genotypes and gene expression; it is supported by the Common Fund of the Office of the Director of the National Institutes of Health as well as by NCI, NHGRI, NHLBI, NIDA, NIMH, and NINDS. All GTEx data retrieved for this study contains no participant data and adheres to the NIH Genomic Data Sharing guideline. We retrieved the processed gene expression V8 dataset directly from the GTEx portal website (https://www.gtexportal.org/home/). In the open access data download page, there are 54 bulk tissue expression files within the “Gene TPMs by tissue” section which provides RNA-seq TPMs information by individual tissues (e.g. gene_tpm_2017-06-05_v8_adipose_subcutaneous.gct.gz). We only used the bulk tissue expression information for this study; therefore, we excluded the gene_tpm_2017-06-05_v8_cells_cultured_fibroblasts and gene_tpm_2017-06-05_v8_cells_ebv-transformed_lymphocytes files. Finally, we started with 52 tissue expression datasets. In the initial summary, we listed the numbers of samples included in each tissue types (Supplementary Table 1). There are 16,704 samples in total. The top abundant tissues are muscle_skeletal (803); whole_blood (755) and skin_sun_exposed_lower_leg (701). On the contrary, there are tissues with limited numbers of donor samples: kidney_medulla (4); fallopian_tube (9); cervix_ectocervix (9) and cervix_endocervix (10). The GTEx sample attribute information was then obtained with the GTEx_Analysis_v8_Annotations_SubjectPhenotypesDS file. We processed the age and sex features for each sample by using the GTEx sample ID.

The HPA (Human Protein Atlas) is a rich resource for the human protein-coding gene expression and their pathological implications^31^. We retrieved the normal tissue expression information from the HPA Data release web site (https://www.proteinatlas.org/about/download). The rna_tissue_hpa.tsv file and rna_tissue_hpa_description.tsv were processed. There are 20,162 genes and 40 tissue types in this dataset. The tissues are: cerebral cortex; choroid plexus; liver; gallbladder; pancreas; salivary gland; esophagus; stomach; kidney; urinary bladder; testis; epididymis; prostate; seminal vesicle; breast; cervix; endometrium; fallopian tube; ovary; placenta; adipose tissue; skin; bone marrow; thyroid gland; parathyroid gland; adrenal gland; lung; heart muscle; skeletal muscle; smooth muscle; tongue; duodenum; rectum; colon; small intestine; spleen; tonsil; lymph node; thymus; appendix. We excluded placenta for subsequent comparison analysis in order to use only the adult tissue types as GTEx project; and about 21 tissues matched with 27 major tissue subtypes utilized in our final analysis.

### Protein-coding genes in the GTEx V8 datasets

The GTEx V8 dataset utilized the GENCODE V26 annotation attributes for their gene expression pipelines. We further obtained the GENCODE V26 attributes form the GENCODE project (https://www.gencodegenes.org). We used the basic gene annotation file: gencode.v26.basic.annotation.gff3. Initially, there are 56,200 gene records in the GTEx retrieved gene expression datasets, there are duplicated gene records in the retrieved gene records and we first removed 44 duplicated gene records with the “PAR-Y” chromosome locations and keep those gene records with only X-chromosome locations. There are 56,156 gene records remained. According to the GENCODE biotype tag, there are 19,273 human protein-coding genes and 36,883 non-coding genes. Thus, we separated the protein-coding genes and non-coding genes. We utilized 19,273 protein-coding genes from 52 tissue types to initialize this study. Later, we used the expression TPM average from 52 tissues for excluding lowly expressed protein-coding genes, and 870 genes were removed for subsequent analyses.

In the final phase of this study, we only selected major representative tissue subtypes with abundant sample size in each tissue (sample numbers referred in Supplementary Table 1). The 27 tissues are: adipose_subcutaneous; adrenal_gland; artery_aorta; bladder; brain_cerebellum; brain_cortex; breast_mammary_tissue; colon_transverse; esophagus_mucosa; heart_ atrial_appendage; liver; lung; minor_salivary_gland; muscle_skeletal; nerve_tibial; ovary; pancreas; pituitary; prostate; skin_sun_exposed_lower_leg; small_intestine_terminal_ileum; spleen; stomach; testis; thyroid; uterus; vagina. The total samples from these 27 tissues are 9,943 in this subset; and the protein-coding genes (with average TPM expression value above 0.05) analyzed were 18,439. All expression and Gini index values were subsequently recalculated in the selected 27 tissue subset here.

### Gini index-subject and Gini index-tissue calculations

Dr. Corrado Gini created the Gini index as a concise indicator of economic inequality in society. It is characterized as the average of the absolute disparities between every pair of individuals for a specific measure (https://github.com/oliviaguest/gini/blob/master/gini.py). The minimal value is 0 when all measurements are equal, and the theoretical maximum is 1. Using Python and NumPy programming tools, we calculated the gene expression averages; coefficient of variation (C.V.); and Gini index to examine the protein-coding gene expression variations from GTEx V8 datasets. The Gini index is often calculated using the mean expression values of distinct tissue types to demonstrate variances in tissue expression. In this context, we employed the phrase “Gini index-TPM”. Consequently, this computation does not consider the variations among donor samples. Hence, we compute the Gini index values for all samples derived from 52 tissue subtypes or the chosen 27 tissue subsets. In this case, we employed the Gini index-subject term to accurately represent the calculations that were performed using all donor samples from combined tissues. Two Gini index values were utilized here to represent the computations from either 52 or 27 tissues in this investigation. Additionally, we are also interested in analyzing the Gini index derived from donor samples within each tissue subtype to investigate the individual variations among different tissue subtypes. We computed the Gini index-tissue values for each individual tissue dataset. Therefore, each protein-coding gene would possess 52 Gini index-tissue values. We also found that there is a high correlation with the Gini index-tissue and C.V. values in tissue subtypes examined (R^2^ = 0.64). In our study, we then determined the average of Gini index-tissues from 52 tissue subtype and compared with the Gini index subject values for each protein-coding gene examined.

### Data processing and graph illustration

Figure graph illustration was performed with the GraphPad Prism (version 10) software package (https://www.graphpad.com). The significance level of P-value was set at the default value of 0.05, as done previously^10,24,42^. The statistical evaluation in Figs. 1 and 4 was conducted using the built-in capabilities within GraphPad Prism. The unpaired t-test was conducted comparing protein-coding genes (19,273) with non-coding genes (36,883) (Fig. 1A,B). The paired t-test was conducted in Fig. 4 to compare the Gini index-subject values and Gini index-tissue values for each of the 18,403 protein-coding genes.

The DAVID (Database for Annotation, Visualization, and Integrated Discovery) functional analysis (https://david.ncifcrf.gov) was used to discover functional enrichment classes. It was used to identify substantially enriched GO keywords and Kyoto Encyclopedia of Genes and Genomes (KEGG) pathways. Pathway enrichment analysis was statistically significant at a P-value < 0.05. Selected genes were uploaded to the DAVID analysis process, which utilized default settings to discover enrichment groups.

### Gene visualization web tool

A visualization database was implemented here using the PHP programming language onto an Apache web server environment in conjunction with the MySQL database. It is hosted in a Docker engine running on an Ubuntu 22.04 Linux server. A JavaScript D3 package is also implemented for graphical display of Gini index values and TPM expression values. All protein-coding gene information are freely accessible at https://hpsv.ibms.sinica.edu.tw.

### Supplementary Information

Supplementary Information 1.

Supplementary Information 2.

## Data Availability

All data examined in this research is included within this publication and its additional supplementary files. The original gene expression datasets were obtained from the GTEx project. The web URL provided allows for the presentation of specific information regarding individual protein-coding genes: https://hpsv.ibms.sinica.edu.tw.
