# Supplementary material for: Housekeeping protein-coding genes interrogated with tissue and individual variations
Source: Sci Rep. 2024 May 30;14:12454. doi: 10.1038/s41598-024-63269-4 (PMC11139953; doi:10.1038/s41598-024-63269-4)

**Supplementary information**

**Housekeeping Protein-coding Genes Interrogated with Tissue and Individual Variations**

Kuo-Feng Tung^1^, Chao-Yu Pan^1^, and Wen-chang Lin^1*^

^1^Institute of Biomedical Sciences, Academia Sinica, Taipei, Taiwan, R.O.C.

Supplemental Table 1. Numbers of samples in 52 tissue subtypes. The GTEx V8 datasets were retrieved from GTEx website and the Gini index-tissue values were determined for protein-coding genes in each tissue subtypes as described in the Methods.

| **GTEx_tissue_subtypes** | **Numbers of GTEx donor sample** | **Average Gini index-tissue values** | **Genes with Gini index  value<0.2** |
| --- | --- | --- | --- |
| adipose_subcutaneous | 663 | 0.32 | 8283 |
| adipose_visceral_omentum | 541 | 0.35 | 5794 |
| adrenal_gland | 258 | 0.33 | 7433 |
| artery_aorta | 432 | 0.31 | 8728 |
| artery_coronary | 240 | 0.34 | 7380 |
| artery_tibial | 663 | 0.33 | 8291 |
| bladder | 21 | 0.32 | 7392 |
| brain_amygdala | 152 | 0.38 | 373 |
| brain_anterior_cingulate_cortex_ba24 | 176 | 0.35 | 1991 |
| brain_caudate_basal_ganglia | 246 | 0.35 | 1156 |
| brain_cerebellar_hemisphere | 215 | 0.30 | 7933 |
| brain_cerebellum | 241 | 0.27 | 10525 |
| brain_cortex | 255 | 0.30 | 7530 |
| brain_frontal_cortex_ba9 | 209 | 0.32 | 3960 |
| brain_hippocampus | 197 | 0.39 | 56 |
| brain_hypothalamus | 202 | 0.38 | 255 |
| brain_nucleus_accumbens_basal ganglia | 246 | 0.37 | 674 |
| brain_putamen_basal_ganglia | 205 | 0.36 | 604 |
| brain_spinal_cord_cervical_c-1 | 159 | 0.37 | 972 |
| brain_substantia_nigra | 139 | 0.37 | 675 |
| breast_mammary_tissue | 459 | 0.36 | 6145 |
| cervix_ectocervix | 9 | 0.31 | 7356 |
| cervix_endocervix | 10 | 0.28 | 9500 |
| colon_sigmoid | 373 | 0.32 | 8065 |
| colon_transverse | 406 | 0.36 | 5055 |
| esophagus_gastroesophageal_junction | 375 | 0.33 | 7693 |
| esophagus_mucosa | 555 | 0.32 | 7278 |
| esophagus_muscularis | 515 | 0.32 | 8072 |
| fallopian_tube | 9 | 0.29 | 8676 |
| heart_atrial_appendage | 429 | 0.35 | 4662 |
| heart_left_ventricle | 432 | 0.42 | 19 |
| kidney_cortex | 85 | 0.41 | 13 |
| kidney_medulla | 4 | 0.25 | 8702 |
| liver | 226 | 0.37 | 3771 |
| lung | 578 | 0.33 | 6539 |
| minor_salivary_gland | 162 | 0.32 | 7435 |
| muscle_skeletal | 803 | 0.39 | 3319 |
| nerve_tibial | 619 | 0.28 | 9745 |
| ovary | 180 | 0.33 | 7979 |
| pancreas | 328 | 0.34 | 5711 |
| pituitary | 283 | 0.30 | 8642 |
| prostate | 245 | 0.34 | 6823 |
| skin_not_sun_exposed_suprapubic | 604 | 0.31 | 7960 |
| skin_sun_exposed_lower_leg | 701 | 0.30 | 8236 |
| small_intestine_terminal_ileum | 187 | 0.36 | 4574 |
| spleen | 241 | 0.30 | 8415 |
| stomach | 359 | 0.38 | 3642 |
| testis | 361 | 0.20 | 11353 |
| thyroid | 653 | 0.30 | 8898 |
| uterus | 142 | 0.32 | 8119 |
| vagina | 156 | 0.35 | 6172 |
| whole_blood | 755 | 0.50 | 35 |

**Figure Legends**

**Supplementary Figure 1.** Different types of Gini index distribution of human protein-coding genes. The box-and-whisker plot demonstrates Gini index-subject values (blue color); Gini index-TPM values (red color) and average Gini index-tissue values (green color) for protein-coding genes. The calculation of Gini index values is described in the Methods section. Statistical assessment is performed by RM one-way ANOVA test. ****: P-value<0.0001.

Supplementary Figure 1


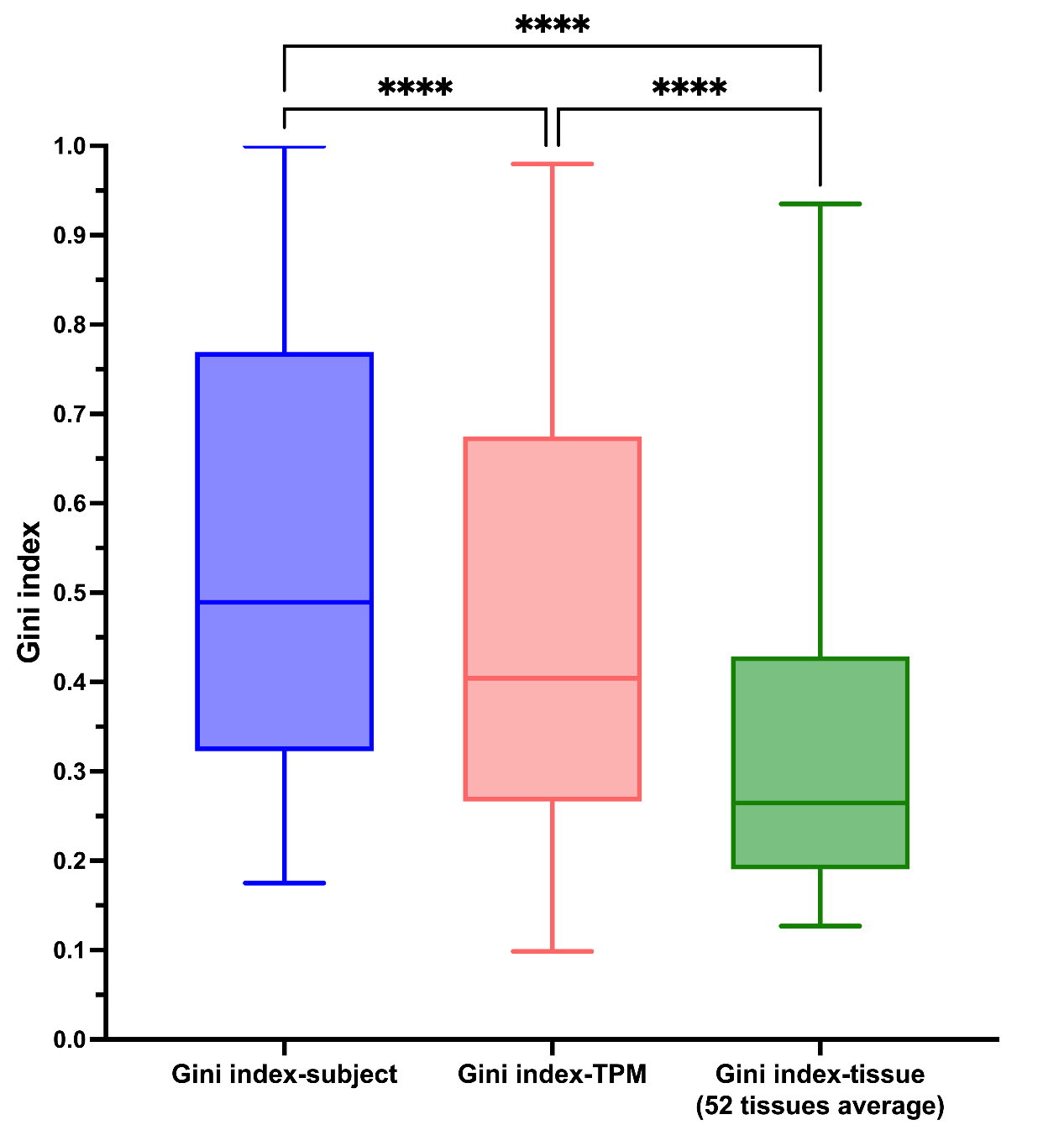


**Supplementary Figure 2.** Illustration of Gini index and gene expression information webpage for PZP Alpha-2-Macroglobulin Like (PZP) protein-coding gene. The information provided includes the fundamental information of the PZP protein-coding gene, such as its IDs and Gini index-subject. On the top panel, there is a visualization graph for the Gini index-tissue, Gini index-tissue(male), and Gini index-tissue(female). To activate or deactivate the display of the Gini index lines, simply click on the symbols. The gene expression information for the TPM is presented on the bottom panel. It is noted that the Gini index calculation for males and females do not include 7 specific tissue subtypes: bladder, ovary, prostate, testis, uterus, and vagina.

Supplementary Figure 2


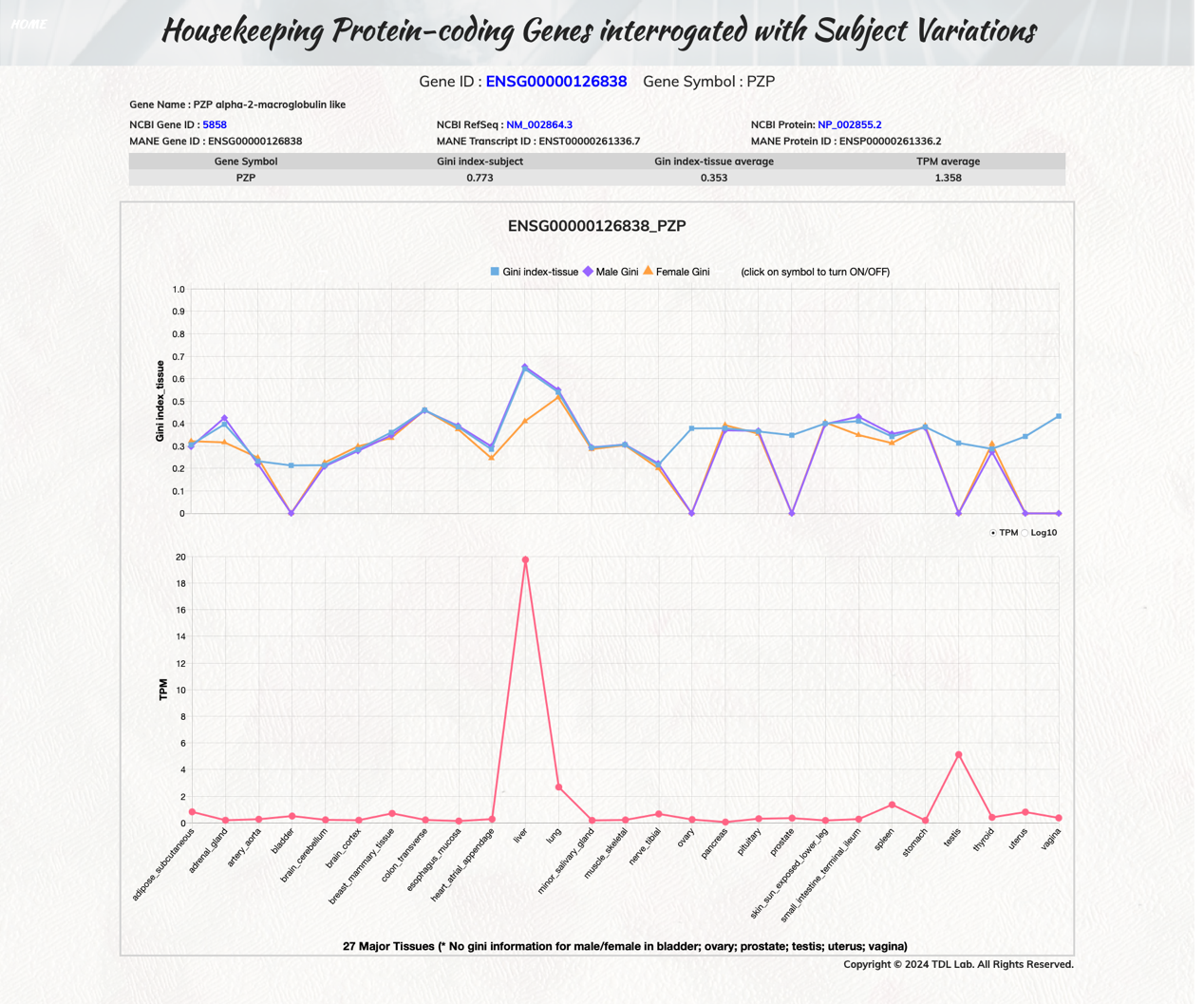

Supplement: Supplementary file 2 — Supplementary Information 2. [file 41598_2024_63269_MOESM2_ESM.docx]
